# Development and psychometric validation of the Nausea/Vomiting Symptom Assessment patient-reported outcome (PRO) instrument for adults with secondary hyperparathyroidism

**DOI:** 10.1186/s41687-018-0029-6

**Published:** 2018-02-13

**Authors:** Colleen A. McHorney, Mark E. Bensink, Laurie B. Burke, Vasily Belozeroff, Chad Gwaltney

**Affiliations:** 1 Evidera, 7101 Wisconsin Avenue, Suite 1400, Bethesda, MD 20814 USA; 2 Amgen Inc., Thousand Oaks, CA USA; 3 University of Maryland, Baltimore, MD USA; 4 Brown University, Providence, RI USA; 5 ERT, Pittsburgh, PA USA

**Keywords:** NVSA^©^, Nausea, Vomiting, Patient-reported outcomes, Qualitative research, Qualitative concept elicitation, Qualitative cognitive interviews, Psychometrics, Chronic kidney disease, Secondary hyperparathyroidism

## Abstract

**Background:**

We developed the Nausea/Vomiting Symptom Assessment (NVSA^©^) patient-reported outcome (PRO) instrument to capture patients’ experience with nausea and vomiting while on calcimimetic therapy to treat secondary hyperparathyroidism (SHPT) related to end-stage kidney disease. This report summarizes the content validity and psychometric validation of the NVSA^©^.

**Methods:**

The two NVSA^©^ items were drafted by two health outcomes researchers, one medical development lead, and one regulatory lead: it yields three scores: the number of days of vomiting or nausea per week, the number of vomiting episodes per week, and the mean severity of nausea. An eight-week prospective observational study was conducted at ten dialysis centers in the U.S. with 91 subjects. Criterion measures included in the study were the Functional Living Index-Emesis, Kidney Disease Quality of Life Instrument, EQ-5D-5 L, Static Patient Global Assessment, and Patient Global Rating of Change. Analyses included assessment of score distributions, convergent and known-groups validity, test-retest reliability, ability to detect change, and thresholds for meaningful change.

**Results:**

Qualitative interviews verified that the NVSA^©^ captures relevant aspects of nausea and vomiting. Patients understood the NVSA^©^ instructions, items, and response scales. Correlations between the NVSA^©^ and related and unrelated measures indicated strong convergent and discriminant validity, respectively. Mean differences between externally-defined vomiting/nausea groups supported known-groups validity. The scores were stable in subjects who reported no change on the Patient Global Rating of Change indicating sufficient test-retest reliability. The no-change group had mean differences and effect sizes close to zero; mean differences were mostly positive for a worsening group and mostly negative for the improvement group with predominantly medium or large effect sizes. Preliminary thresholds for meaningful worsening were 0.90 days for number of days of vomiting or nausea per week, 1.20 for number of episodes of vomiting per week, and 0.40 for mean severity of nausea.

**Conclusions:**

The NVSA^©^ instrument demonstrated content validity, convergent and known-groups validity, test-retest reliability, and the ability to detect change. Preliminary thresholds for minimally important change should be further refined with additional interventional research. The NVSA^©^ may be used to support study endpoints in clinical trials comparing the nausea/vomiting profile of novel SHPT therapies.

## Background

Chronic kidney disease (CKD) is a progressive loss in renal function. Secondary hyperparathyroidism (SHPT) is a frequently-encountered problem in CKD with resulting impact on the morbidity and survival of hemodialysis patients [1–7]. Several therapeutic strategies have been used to manage SHPT including the oral calcimimetic Sensipar® (cinacalcet) [8]. The most frequently-reported adverse reactions associated with cinacalcet have been nausea and vomiting [8–10]. These side effects may adversely affect medication adherence, therapeutic efficacy, and patients’ clinical and quality-of-life outcomes. A new generation of calcimimetic is being developed as an intravenous formulation. This innovation may improve patients’ calcimimetic nausea/vomiting experience; however, an instrument tailored to daily nausea and vomiting assessment would be needed to quantify the impact.

We developed the Nausea/Vomiting Symptom Assessment (NVSA^©^) patient-reported outcome (PRO) instrument to capture the number of days of nausea or vomiting per week, number of episodes of vomiting per week, and mean severity of nausea among CKD patients on maintenance hemodialysis with SHPT. The US Food and Drug Administration (FDA) PRO Guidance document [11] states that PRO measures should be conceptually valid as they relate to the medical intervention and disease state being studied, meet a threshold of psychometric soundness, and be relevant to patients. Similarly, the Committee for Medicinal Products for Human Use reflection paper [12] states “the use of specific [health-related quality of life] HRQoL domains as study endpoints pre-supposes that the HRQoL instrument was adequately developed and fully validated prior to measuring the subset of domains chosen.”

The NVSA^©^ was developed because existing measures of nausea/vomiting do not harmonize the concepts of interest (nausea/vomiting) with the context of use (comparative studies of new therapies for CKD patients with SHPT) [13]. Some existing measures are dictotomous in format or have long recall periods [14], thus potentially yielding imprecise information on change in the frequency and severity of nausea/vomiting due to therapy. Still others assess the impact of nausea/vomiting symptoms on HRQoL [15, 16] instead of nausea/vomiting themselves. Thus, the NVSA^©^ was developed to provide a direct, focused, daily measure of nausea and vomiting in comparative studies of SHPT therapies.

PRO instruments designed to assess treatment-related adverse reactions come with unique challenges in their development and validation. For example, the sample of patients used in psychometric testing must have the underlying target illness but must also be using a treatment that results in the adverse reaction. Because not all patients using the target treatment will experience adverse reactions, it can be difficult to ensure adequate variability―both between and within patients―in a study sample. Further, for PRO instruments that assess treatment-related adverse reactions, assessment of clinically-meaningful change should be focused on onset or worsening of symptoms over time. In other words, patients in a comparative trial that includes treatment-related adverse reactions as a study endpoint would likely start with no or minimal symptoms―some patients would then develop symptoms or experience worsening in treatment-related symptoms during the study. Rather than identifying the definition of a meaningful response to treatment, there must be an a priori identification of a meaningful worsening in the severity of reactions. We addressed these unique aspects of treatment-related PRO development herein by describing the development and psychometric evaluation of the NVSA^©^.

## Methods

### Study design

Overview of NVSA^©^ Development and Validation.

**Fig. 1:**
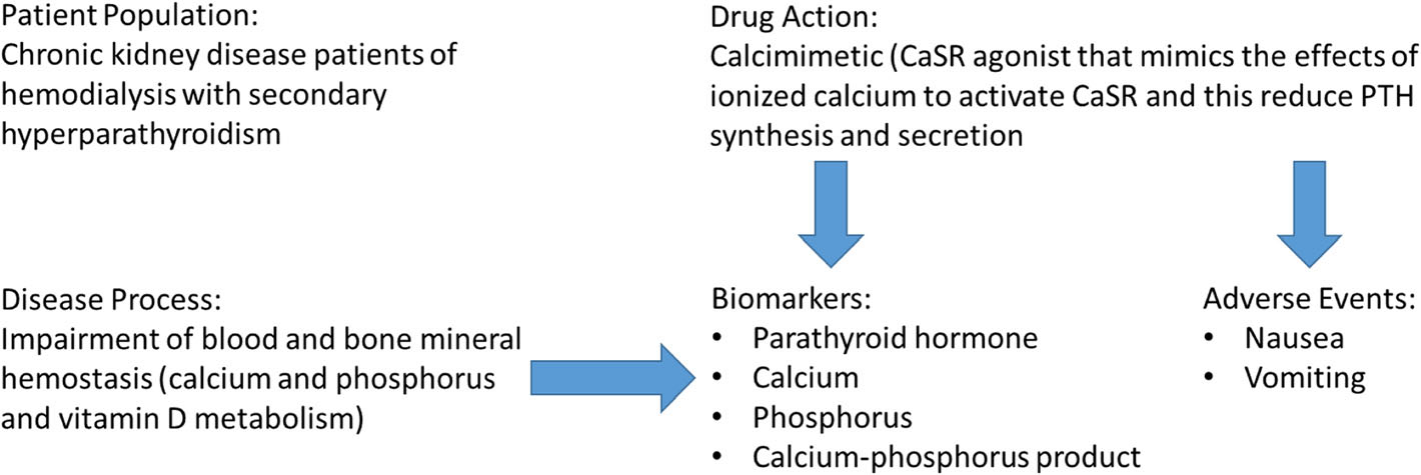
Disease Model for the NVSA^©^ Development

As the FDA PRO Guidance document [11] notes, a “fundamental consideration in the review of a PRO instrument is the adequacy of the item generation process to support the final conceptual framework for the instrument.” However, as the guidance also outlines, **“**in some cases, the question of what to measure may be obvious given the condition being treated.” A review of information from clinical studies [8–10, 17–19] and expert clinical guidelines [20] supported nausea and vomiting as important adverse reactions associated with the oral calcimimetic cinacalcet. Given the evidence identifying calcimimetic treatment-emergent nausea and vomiting as a recognized limitation of cinacalcet [8–10, 17–20], the two NVSA^©^ items focused on these two important adverse reactions. The items were developed based on a review of data from cinacalcet trials, discussions with clinical SHPT experts, a review of PRO measures that assess nausea/vomiting, and initial hypotheses regarding the endpoints that would be most appropriate for use in future efficacy trials of novel (i.e., non-cinacalcet) trials for SHPT. The disease model for the NVSA^©^, which guided PRO development, is presented in Fig. 1.

Because measuring vomiting and nausea was obvious for the purpose of PRO development, the initial items and response categories were drafted by a cross-functional expert team that included two health outcomes/PRO researchers, one medical development lead, and one safety/regulatory lead. Based on the above premises, particularly the FDA PRO Guidance [11], traditional hypothesis-generating concept-elicitation interviews were not executed but were exchanged instead with qualitative cognitive-debriefing interviews to document that patients could understand the NVSA^©^ instructions, items, and response scales and could easily comprehend and meaningfully respond to the NVSA^©^ items. Confirmatory, qualitative concept-elicitation interviews were conducted to provide final, supportive evidence of the importance of the concepts of nausea and vomiting for patients who experienced these adverse reactions associated with oral calcimimetic cinacalcet. The psychometric findings, using data from an observational study dedicated to assessment of the NVSA^©^, were designed to document the measurement properties of the NVSA^©^ as a PRO that can be used to measure nausea/vomiting in clinical trials involving the specific population of interest―CKD patients on maintenance hemodialysis with SHPT.

#### Phase I: Cognitive-debriefing interviews

Phase I utilized individual, face-to-face interviews to assess patient understanding and ease of self-administration of the draft NVSA^©^ items on an electronic diary (eDiary). Recruitment targeted 12 adult CKD patients on maintenance hemodialysis with SHPT. A quota was implemented to have 50% (*n* = 6) of the 12 subjects be currently treated with cinacalcet at the time of screening. Two clinical sites enrolled patients (Houston, TX and Washington, DC). Clinicians were oriented to the study procedures, protocol, and requirements over the telephone and were asked to screen and recruit patients and schedule them for an interview if they qualified. Potential patients were telephoned and screened for eligibility against study criteria. Those eligible were asked to attend an enrollment and interview visit at the site to review and sign the Informed Consent Form, complete the Demographic Form, and to participate in an interview. Interviewers conducted in-person, one-on-one interviews using a semi-structured cognitive-interview guide. The interviews were conducted in two separate waves of six subjects each. Individual interview sessions lasted approximately 30 min. Institutional Review Board (IRB) submission and approval from Quorum Review IRB for the cognitive-interview study was secured by DaVita Clinical Research who maintained appropriate documentation and conducted management of the study in accordance with the ethical principles consistent with Good Clinical Practice and applicable regulatory requirements.

#### Phase II: Confirmatory concept-elicitation interviews

Phase II included adult subjects with CKD on dialysis who were receiving treatment for SHPT and who participated in the eight-week, prospective, observational study. Adult patients were recruited from six DaVita dialysis centers in the U.S. in California, Kansas, Connecticut, and Michigan. Subjects who experienced nausea/vomiting during the psychometric observational study were identified by eResearch Technologies (ERT) from NVSA^©^ diary records. This information was communicated to the DaVita primary contact and the clinical site where that subject’s study enrollment was managed. Staff at the clinic sites initiated recruitment communications with the subjects, conducted the consent process, and scheduled the telephone interview. Each individual interview lasted approximately 60 min. A total of ten subjects were enrolled. IRB submission and approval from Quorum Review IRB for the interviews was secured by DaVita Clinical Research who maintained appropriate documentation and conducted management of the study in accordance with the ethical principles consistent with Good Clinical Practice and applicable regulatory requirements.

#### Phase III: Psychometric validation

This was an eight-week, prospective, observational study. The eight-week time period was selected based on the timing of incident nausea/vomiting in clinical trials [8, 18, 19] as well as anecdotal information from the real-world experiences of physicians prescribing cinacalcet. To capture the full range of experiences of nausea/vomiting, three cohorts of subjects were recruited: (1) those who started cinacalcet in the month before enrollment (‘Initiating Cinacalcet’ cohort); (2) those who had SHPT but had no history of previous treatment with cinacalcet (‘Cinacalcet Naïve’ cohort); and (3) those who were current users of cinacalcet and initiated cinacalcet greater than 1 month prior to enrollment (‘Variable Time on Cinacalcet’ cohort). Subjects were recruited into each cohort in a 1:1:2 ratio to enroll a sample with adequate variability in nausea/vomiting.

Subjects were enrolled if they were at least 18 years of age and had maintenance hemodialysis three times per week for at least 3 months prior to enrollment. Subjects must have had: (a) SHPT defined as two consecutive laboratory serum parathyroid hormone values > 400 pg/mL without cinacalcet; or (b) had initiated cinacalcet in the last month; or (c) been currently using cinacalcet for greater than 1 month at the time of enrollment. Subjects were excluded if they were not able to give written informed consent and/or comply with the study procedures, if they had any other illness (with the exception of diabetes) that causes nausea/vomiting and that might confound subjects’ responses to interview questions, if they were receiving chronic antiemetic therapy, if they were pregnant or lactating, or if they were currently participating in another investigational device or drug study.

A total of 91 subjects were recruited across ten dialysis centers in the US. IRB submission and approval from Quorum Review IRB for the study was secured by DaVita Clinical Research who maintained appropriate documentation and conducted management of the study in accordance with the ethical principles consistent with Good Clinical Practice and applicable regulatory requirements.

### Measures

#### Phase I: Cognitive-debriefing interviews

Cognitive interviewing is a method of testing a PRO instrument to identify: comprehensibility of the language, format, and instructions; appropriateness of response options; and overall feasibility and burden [21]. A cognitive-interview guide facilitated the cognitive assessment (patient comprehension) of the draft NVSA^©^ items. The cognitive-interview guide used a process requiring patients to “think aloud” where patients were asked to respond to a specific set of questions regarding the instructions, questions, and response options. The “think aloud” technique allowed patients to verbalize the thought process involved in providing a response to the questions in the NVSA.^©^ During the interview, patients self-administered the NVSA^©^ and answered questions about their comprehension of the items. Two primary components were tested: (1) the intent of the question (what the respondent believes the question is asking), and (2) the meaning of terms (what specific words and phrases in the question mean to the respondent). The information gathered during the individual interviews was used to inform revisions to the content of the eDiary and to create patient training for the administration of the NVSA.^©^ The primary focus of the cognitive interview was on the items themselves rather than on administration procedures. Other questions in the cognitive interviews focused on the response option and instructions and further examined specific terminology to ensure patients understood the NVSA^©^ items. At the close of the interview, patients completed an “Ease of Use Questionnaire” to report their experience regarding the usability of the eDiary.

#### Phase II: Confirmatory concept-elicitation interviews

The confirmatory concept-elicitation interviewers used a semi-structured interview guide to explore patient perceptions about their experience with nausea/vomiting associated with treatment for SHPT. The semi-structured qualitative interview guide included open-ended questions and a day-reconstruction exercise to invite spontaneous responses from subjects regarding treatment-related nausea/vomiting. Each interview focused first on issues more proximal to treatment-related nausea/vomiting (symptoms) and then moved to more distal issues (impacts). All interviews were conducted using a process that began with open-ended questions before moving to follow-up probe questions for areas not initially mentioned by subjects. A series of follow-up probing questions were also included to explore sign, symptom, and impact language and areas not mentioned spontaneously by subjects. Worksheets were used to obtain numeric ratings of characteristics of the elicited concepts that subjects experienced.

#### Phase III: Psychometric validation

The NVSA^©^ is a two-item PRO instrument designed to assess nausea/vomiting on a daily basis. An eDiary was used to administer the NVSA^©^ each evening. The first item asks about severity of nausea in the past 24 h using a 0–10 numeric rating scale (no nausea to nausea as severe as you can imagine). The second item asks about the number of times the subject vomited in the past 24 h using a “spinner control” to select a number between zero and 99.

External validity criteria for the psychometric evaluation included the Functional Living Index-Emesis (FLIE-Emesis) [22], Kidney Disease Quality of Life Instrument (KDQOL-36™) [23], EQ-5D-5 L [24], Static Patient Global Assessment (sPGA) measure, and a Patient Global Rating of Change (PGRC).

The FLIE-Emesis is an 18-item PRO instrument that measures the impact of nausea and vomiting on physical activities, social and emotional function, and ability to enjoy meals over the past 5 days [22]. The FLIE contains nine items for nausea and nine for vomiting. The total score ranges from 18 to 126 with higher scores indicating a less negative impact on patients’ daily life due to nausea and vomiting. Two subscales can be derived: FLIE vomiting and FLIE nausea (both have a score range of 9 to 63).

The KDQOL-36™ is a 36-item PRO instrument specific to CKD with a four-week recall [23]. For the psychometric evaluation, three KDQOL-36™ single-item measures were used: bothersomeness of nausea, faintness, and shortness of breath. Each item yields scores ranging from 1 (not at all bothered) to 5 (extremely bothered).

The EQ-5D-5 L is a generic measure of health status that provides descriptive profiles and a single-index value. [24] The recall period is “today” (at the time of completion). The five EQ-5D-5 L descriptive profiles and its visual analogue scale (VAS) measuring health today were used to assess the discriminant validity of the NVSA^©^.

sPGA (Static Patient Global Assessment) – Nausea and Vomiting is a two-item measure in which subjects indicate an overall assessment of current nausea and vomiting. Response choices include no symptoms, very mild, mild, moderate, severe, and very severe symptoms. The nausea and vomiting items were scored separately.

PGRC (Patient Global Rating of Change) – Nausea and Vomiting is a two-item measure in which subjects indicate worsening or improvement in nausea and vomiting since the onset of the study. Response choices include very much improved, much improved, minimally improved, no change, minimally worse, much worse, and very much worse. The nausea and vomiting items were scored separately.

Patients were trained to use the eDiary upon enrollment. The NVSA^©^ was completed on the eDiary at home each evening during the study. Subjects completed the FLIE, KDQOL-36,™ EQ-5D-5 L, sPGA, and PGRC on eDiaries before dialysis during the enrollment visit. Follow-up FLIE and PGRC and sPGA measures were completed weekly thereafter, also before dialysis at one of the subject’s three times per week dialysis clinic visits, for the duration of the study. Follow-up KDQOL-36™ and EQ-5D-5 L measures were completed at the week 4 and week 8 dialysis clinic visit.

### Analyses

#### Cognitive-debriefing interviews

The cognitive interviews were audio-recorded. After each wave of cognitive interviews, the interviewer wrote a description of problematic cognitive issues for each item if any were apparent. The description of potential issues, along with recommendations for retaining, modifying, or eliminating an item, were discussed with the development team and, following agreement, changes were made to the draft items for the second wave of cognitive interviews. After the second wave of interviews, the results were again reviewed by the development team and recommendations for the items were documented. The transcripts were reviewed in order to extract quotations from the patients’ responses to each question asked in the cognitive-interview guide. Quotations were summarized to show patient interpretation and understanding of each item and its response option and to identify any difficulties that arose with the proper understanding of the item content.

#### Confirmatory concept-elicitation interviews

The confirmatory concept-elicitation interviews were audio recorded. The digital audio files of subject interviews were transcribed by a professional transcription company. Transcripts were coded using ATLAS.ti software to organize coded concepts by similarity of content. The primary goal of transcript coding was to organize and catalog subject descriptions of their treatment-related nausea/vomiting symptoms. The coding framework provided an organization for grouping like-concept codes. As the coding process continued, the coding framework was expanded by adding concepts that subjects identified in the interview process. Saturation of concepts was assessed across four grouping of the ten transcripts: (1) first to third interview; (2) fourth to sixth interview; (3) seventh to eighth interview; and (4) nine to tenth interview.

#### Psychometric validation

Standard psychometric techniques, consistent with those recommended by the FDA [11], were used to assess the measurement properties of the NVSA^©^.

### Handling of data

Mean severity of nausea was calculated by averaging all available daily severity ratings. No imputation was performed for missing responses. For psychometric evaluation, daily severity data were averaged into a weekly (i.e., seven day) score.

The number of episodes of vomiting per week was estimated as the total number of episodes in a given week. Consistent with other diary-based PRO research [25–31], if a subject was missing valid responses on three or fewer days in a week, the available valid responses in the week were used to estimate the total number of episodes of vomiting equivalent to a seven-day week. If the subject had missing responses for four or more days for a given week, data from that week were considered missing [32, 33].

Number of days of vomiting or nausea per week was derived from the first and second item. If a subject provided a valid response to the severity of nausea question that was > 0 but did not provide a valid response to the vomiting-episode question, this day was counted as a day of nausea/vomiting. If a subject provided a valid response to the vomiting-episode question that was > 0 but did not provide a valid response to the nausea-severity question, this day was counted as a day of nausea/vomiting. If the answer to the number of vomiting episodes was 99 (the maximum allowable value on the eDiary spinner), then the data were considered missing.

Descriptive statistics are presented for each of the 8 weeks of data collection. Tests of validity (convergent, discriminant, and known groups) involve week 1 data. Test-retest reliability used week 1 and week 2 data. Sensitivity to change and anchor-based assessment of meaningful change involved weekly data. All analyses were conducted using SAS Version 9.2.

### Item-level score distributions

Item-level distributional statistics were tabulated including mean, median, standard deviation (SD), percentage of the sample at the floor (score of 0―lowest possible score―or the least possible nausea/vomiting) and ceiling (highest possible score or the greatestt possible nausea/vomiting), and item-level missing data. Floor and ceiling effects were considered to be present if they exceeded 15% [34].

### Convergent validity

The NVSA^©^ scores were hypothesized to correlate significantly and moderately with criterion measures assessing nausea/vomiting (convergent validity with the FLIE total, nausea, and vomiting scores and KDQOL-36™ nausea bothersomeness) but have low correlations with criterion measures assessing unrelated symptoms (discriminant validity with the KDQOL-36™ shortness of breath and faintness bothersomeness and EQ-5D-5 L). Convergent and discriminant validity were assessed with Spearman correlations due to the non-normal nature of the NVSA^©^distributions. A strong correlation was defined as a Spearman correlation ≥ 0.50, moderate correlation as ≥ 0.30 but < 0.50, and weak correlation as <  0.30 [35].

### Known-groups validity

Known-groups validity compared NVSA^©^ scores across groups that differed in their nausea/vomiting as defined by criteria external to the NVSA^©^. Subjects reporting ‘no symptoms’ on the sPGA were compared to subjects who reported any level of symptoms. Subjects reporting being ‘not at all bothered’ on the KDQOL-36™ nausea item were compared to subjects who reported any level of nausea bothersomeness. Subjects who had a FLIE total score equal to or higher than 108 (indicating no or minimal nausea/vomiting) were compared to subjects with scores lower than 108 (indicating presence of nausea/vomiting) [36, 37]. The Wilcoxon two-sample test was used to assess known-groups validity.

### Test-retest reliability

A stable subgroup was defined as those who experienced no change in nausea/vomiting as measured by the PGRC. The intraclass correlation coefficient (ICC) was derived from a repeated measures, two-way, random effects model. The ICC was calculated from week 1 to week 2. ICC’s of 0.70 or higher are considered acceptable [38, 39].

### Ability to detect change

Ability to detect change (or responsiveness) is an aspect of longitudinal construct validity [40, 41]. Ability to detect change can be assessed by evaluating the relationship between changes in the PRO of interest to changes in external criteria (anchors) whether they be clinical or other patient-reported criteria [42]. Once responsiveness has been documented, range of values for thresholds for meaningful change can be estimated. The weekly sPGA was used as the external anchor in tests of ability to detect change. Sample size for the any improvement and any worsening groups had to be at least *n* = 6 for the analysis to be executed. sPGA nausea was the criterion for number of days of vomiting or nausea and mean severity of nausea. sPGA vomiting was the criterion for number of episodes of vomiting. Per well-established recommendations, the weekly NVSA© change scores were correlated (Spearman correlations) with their weekly anchor sPGA change score to assess the strength of the relationship between the anchor and the PRO instrument. It is recommended that anchor-PRO instrument correlations be 0.30–0.37 [42, 43]. Within-patient change scores (e.g., week 2-week 1, week 3-week 2) were generated and were summarized with within-patient effect sizes [44]. The within-patient effect size was defined as the within-patient change score (post-pre score) divided by pre (group) standard deviation. Because of the small sample size and the sparse distribution of the PGRC for worsening, the PGRC could not be used as an external anchor.

### Distribution-based and anchor-based assessments of minimal meaningful change

Distribution-based methods were 0.50 SD and one standard error of measurement (SEM) [42, 45]. The mean and median of the eight weekly 0.50 SDs generated the 0.50 SD criterion. The SEM was calculated from SD * √1-reliability. The SEM was calculated using the test-retest ICC and the mean SD across the 8 weeks.

The sPGA was used to define minimally improved, no change, and minimally worse nausea/vomiting subgroups for anchor-based estimates. Minimal improvement was defined as a within-patient change score between contiguous sPGAs of − 1.0, minimal worsening was a within-patient change score between contiguous sPGAs of 1.0, and no change was a within-patient change score of zero. Because the sPGA was completed weekly, weekly within-patient change scores (e.g., week 2-week 1, week 3-week 2) were estimated for the minimal-improvement and minimal-worsening groups. Because of the small sample size and the sparse distributions of the PGRC for worsening, the PGRC could not be used as an anchor.

## Results

### Cognitive-debriefing interviews

A total of 12 interviews (six at each site) were scheduled. However, one subject did not show up for the interview, and no other eligible patients were available at that time. Therefore, 11 patients (six in wave 1 interviews [Washington, D.C.] and five in wave 2 interviews [Houston, TX]) were included in the data analysis across two sites. The mean age of the interview patients was 54 years, and the sample was 64% percent male. Just over one half (55%) of the patients were Black or African American. Just over one third (36%) of patients had completed less than a high-school education.

The cognitive interviews revealed that two patients mixed the meaning of nausea and vomiting. For clarity and consistency across patients’ understanding of the meaning of these terms, it was decided to add patient training on the definitions of nausea and vomiting using examples of idiomatic phrases provided by patients. The second wave of interviews showed that the training solved this issue. For the nausea-severity item, four patients preferred an alternate wording for the anchor of “10” (“worst possible nausea” vs. “nausea as severe as you can imagine”). However, with exception of a single patient, patients understood both wordings of the scale, thought they were identical, and could answer the question identically with either response scale. It was decided to retain the original wording (“nausea as severe as you can imagine”) to align with FDA [46] and IMMPACT [47] recommendations on assessment of pain intensity wherein the worst anchor is defined by “pain as bad as you can imagine” as well as single-item ratings scales used in other PRO instruments that define the extremity of the symptom of interest with “as bad as you can imagine.” [27, 48–55] Cognitive interviews in other therapeutic areas also confirmed that patients can understand the response scale of “as bad as you can imagine.” [55] Four patients did not answer items thinking of the specified 24-h recall period. This did not appear to result from wording of the items but from lack of experience with recall period. It was decided to add this point to patient training using a visual aid to clearly explain the 24-h recall period. The second wave of interviews showed that the training solved this issue. Three patients had difficulty using the spinner widget to enter their numerical answer. It was decided that this would be an additional feature for which patients received training. Simple training was not enough to solve this issue for two patients in Wave 2. It was decided to enhance this training with exercises on the use of the spinner. Nearly three quarters (73%) of patients described the eDiary as “very easy” or “quite easy” to use. Two patients (18.2%) reported having difficulty using the eDiary, but 100% of patients described the device as “acceptable to use.”

### Confirmatory concept-elicitation interviews

A total of ten concept-elicitation interviews were completed from six sites from DaVita dialysis centers across the US. The concept-elicitation interviews were completed by four female and six male patients who had experienced nausea/vomiting associated with treatment for SHPT.

Nearly one half (46%) of the coded concepts were identified in the first group (first to third interview), additional 43% were identified in group 2 (fourth to sixth interview), and 7% were identified in group group 3 (seventh to eighth interview), respectively. Only one new concept was identified in the fourth and final group (ninth to tenth interview): a single patient reported cutting down on smoking cigarettes because smoking made the nausea/vomiting worse. Given that this impact is relevant only to the population of patients who smoke tobacco―and its causal link to nausea/vomiting among CKD patients on maintenance hemodialysis with SHPT treated with oral calcimimetics is questionable―it is reasonable to dismiss this particular concept and assume that the ten transcripts demonstrated saturation of concept.

Patients most commonly reported that they started to experience nausea/vomiting relatively soon after starting treatment. In most cases, the symptoms began within 1 or 2 weeks of starting treatment. If they experienced gastrointestinal issues prior to the treatment, it typically became worse after starting treatment. Patients typically described having “nausea” or feeling “nauseous” or “nauseated.” Other terms used to describe the experience were having a “queasy” stomach that “feels bad,” “upset,” or “sick.” When asked how they would describe mild nausea, patients described an episode that was not bothersome or an episode of nausea without the vomiting. Nausea was considered severe if it impacted patients’ daily life. Some reported that severe nausea rendered them unable to walk or “do anything.” Participants were asked to rate the severity of their nausea when the symptom was at its worst (using a 0–10 numerical rating scale with zero indicating “No nausea,” and 10 indicating “Nausea as severe as you can imagine”). On average, the patients rated their nausea at 6.9 out of 10 in severity (SD of 2.8) with a median of 8.0.

Patients distinguished between nausea and vomiting and experienced them as separate, but related, symptoms. While nausea was typically described as a sickly feeling, subjects described “throwing up” or “vomiting” as when this feeling was followed by ejecting the contents of their stomach into their mouth. Episodes of nausea were often, but not always, followed by vomiting. However, subjects frequently described experiencing nausea without vomiting.

While some patients experienced nausea on a daily or near-daily basis, others experienced it only once or twice a month or intermittently. Patients experienced variation in the amount of time they felt nauseous. For some, episodes of nausea were relatively brief lasting 10 to 15 min or less. Others described feeling nauseous for an entire day. When patients were asked to explain how often they vomit, most reported the number of vomiting episodes they experienced per day as opposed to per week or per month. Frequency of vomiting ranged from once or twice a month or “rarely” to up to eight or nine times in 1 day. Patients were also asked to estimate the number of times they vomit in a 24-h period when they experience vomiting at its worst. Seven out of ten patients responded to the question with a mean frequency of 2.4 times per day and answers ranged from once per day to eight times per day. Vomiting episodes were typically counted as the number of times a patient’s body ejected the contents from their stomach into their mouth. Each episode of vomiting was counted individually even if multiple episodes happened within a short timeframe.

### Psychometric validation

#### Cohort description

As shown in Table 1, subject age ranged from 25 to 83 with a mean of 53.5 and a median of 54. Males constituted 59% of the sample. The racial composition of the sample included 36% white, 59% of black or African American, 2% Asian, and 2% American Indian or Alaskan native. One fifth (19%) of the sample was of Hispanic or Latino heritage. One quarter (25%) of the sample was never married, 45% married (or living as married), 14% divorced, and 8% each separated or widowed. Mean years of education was 13.1 with 9% reporting less than a high-school education, 42% a high-school education, 35% some college, and 14% a college education. In terms of employment, 38% reported being disabled/unable to work, 23% were retired, 21% were unemployed, 16% were employed either part-time or full-time, and 2% were homemakers. Almost two thirds of the sample (64%) reported an annual income of less than $15,000; 13% reported an income between $15,000–$25,000, 6% between $25,000–35,000, 10% between $35,000–$50,000, and 7% greater than $50,000. Just over one half of the sample (53%) were ‘Variable Time on Cinacalcet,’ 33% were ‘Cinacalcet Naïve,’ and 14% were ‘Initiating Cinacalcet.’

**Table 1:** Demographic and clinical characteristics of subjects

	*N* = 91
Age	
Mean (SD)	53.5 (12.7)
Median (range)	54 (25–83)
Gender	
Male	59%
Race	
White	36%
Black or African American	59%
Asian	2%
American Indian or Alaskan native	2%
Ethnicity: % Hispanic or Latino	19%
Marital Status	
Never Married	25%
Married (or Living as Married)	45%
Separated	8%
Divorced	14%
Widowed	8%
Education	
Mean Years of Education(SD)	13.1 (2.3)
Less than High School	9%
High School	42%
Some College (13–15 years)	35%
College Education	14%
Employment Status	
Employed Full-Time	9%
Employed Part-Time	7%
Disabled/Unable to Work	38%
Unemployed	21%
Retired	23%
Homemaker	2%
Income	
Less than $15,000	64%
$15,000–$25,000	13%
$25,000–$35,000	6%
$35,000–$50,000	10%
$50,000+	7%
Study cohort	
Variable Time on Cinacalcet	53%
Cinacalcet Naive	33%
Initiating Cinacalcet	14%

#### Descriptive statistics

For each of the NVSA^©^ scores, floor effects (absence of nausea and vomiting) were large (according to well-accepted criteria [34]) at baseline (> 47% across the three NVSA^©^ scores) and increased over the course of the study indicating less nausea/vomiting over time (Table 2). Ceiling effects (greatest severity of nausea and vomiting) were under minimal (according to well-accepted criteria: [34] 10.5% for number of days of vomiting or nausea, under 2.0% for number of episodes of vomiting, and under 3.5% for mean severity of nausea. Missing data rates ranged from a low of 2.5% (week 1) to to a high of 9.9% (week 2). There were no discernible patterns to missing-data rates across the eight study weeks.

**Table 2:** Descriptive Statistics for NVSA^©^ Scores

	N	Mean	Median	SD	% Floor	% Ceiling	% Missing	Test-Retest Reliability^a^
Number of Days of Vomiting or Nausea								0.93
Week 1	80	1.98	1.0	2.46	47.4%	10.3%	2.5%	
Week 2	71	1.85	1.0	2.35	46.9%	9.4%	9.9%	
Week 3	75	1.62	0.0	2.37	56.2%	9.6%	2.7%	
Week 4	70	1.63	0.0	2.34	56.9%	7.7%	7.1%	
Week 5	69	1.55	0.0	2.49	64.2%	9.0%	2.9%	
Week 6	70	1.61	0.0	2.48	55.9%	13.2%	2.9%	
Week 7	68	1.51	0.0	2.52	66.7%	10.6%	2.9%	
Week 8	71	1.23	0.0	2.15	64.6%	6.2%	8.4%	
Number of Episodes of Vomiting								0.61
Week 1	80	0.97	0.0	1.75	69.2%	1.3%	2.5%	
Week 2	71	1.42	0.0	3.88	68.8%	1.6%	9.9%	
Week 3	75	0.70	0.0	1.89	78.1%	1.4%	2.7%	
Week 4	70	0.82	0.0	2.11	78.5%	1.5%	7.1%	
Week 5	69	0.54	0.0	1.50	82.1%	1.5%	2.9%	
Week 6	70	0.83	0.0	2.12	79.4%	1.5%	2.9%	
Week 7	68	0.68	0.0	1.78	80.3%	1.5%	2.9%	
Week 8	71	0.57	0.0	2.34	86.2%	1.5%	8.4%	
Mean Severity of Nausea								0.94
Week 1	80	0.78	0.1	1.24	50.0%	1.3%	2.5%	
Week 2	71	0.80	0.0	1.40	51.6%	1.6%	9.9%	
Week 3	75	0.52	0.0	0.95	56.2%	1.4%	2.7%	
Week 4	70	0.52	0.0	1.04	58.5%	3.1%	7.1%	
Week 5	69	0.51	0.0	1.12	64.2%	1.5%	2.9%	
Week 6	70	0.55	0.0	1.25	55.9%	1.5%	2.9%	
Week 7	68	0.51	0.0	1.00	66.7%	1.5%	2.9%	
Week 8	71	0.38	0.0	0.83	64.6%	1.5%	8.4%	

*N* sample size, *SD* standard deviation

Floor = percentage of the sample obtaining the lowest possible score (score of 0) (absence of nausea and vomiting)

Ceiling = percentage of the sample obtaining the highest possible score (greatest nausea and vomiting)

^a^Test-retest reliability was intraclass correlations between week 1 to week 2

#### Test-retest reliability

The ICCs for week 1 to week 2 were 0.93 for number of days of vomiting or nausea, 0.61 for number of episodes of vomiting, and 0.94 for mean severity of nausea (Table 2).

#### Convergent and Discriminant validity

A total of nine convergent correlations with the FLIE were tested, and 100% were statistically significant (Table 3). Spearman correlations of the NVSA^©^ scores with the FLIE ranged from − 0.38 to − 0.60 (mean and median of − 0.56 and − 0.51, respectively); only two of the nine convergent correlations were less than 0.40. Six of the nine correlations (67%) of the NVSA^©^ with the FLIE met standard criteria for a strong correlation while the remaining three correlations met standard criteria for a moderate correlation. Spearman correlations of the NVSA^©^ scores with the KDQOL-36™ ranged from 0.38 to 0.67 (mean and median of 0.57 and 0.65, respectively), and two of the three correlations (67%) met standard criteria for a strong correlation (Table 4).

**Table 3:** Assessment of convergent validity of NVSA^©^ scores with the FLIE: Week 1

	Convergent validity with the FLIE		
NVSA©	Total score	Nausea subscale	Vomiting subscale
Number of Days of Vomiting or Nausea	s = −0.56 *p* = < 0.0001	s = −0.56 *p* = < 0.0001	s = −0.56 *p* = < 0.0001
Number of Episodes of Vomiting	s = − 0.38 *p* = 0.0007	s = − 0.38 *p* = 0.0006	s = − 0.40 *p* = 0.0003
Mean Severity of Nausea	s = − 0.60 *p* = < 0.0001	s = − 0.60 *p* = < 0.0001	s = − 0.58 *p* = < 0.0001

s = Spearman correlation

**Table 4:** Assessment of convergent and discriminant validity of NVSA^©^ Scores with the KDQOL-36:™ Week 1

	ConvergentValidity	Discriminant Validity	
NVSA^©^	KDQOL-36™ Nausea Bothersomeness	KDQOL-36™ Shortness of Breath	KDQOL-36™ Faintness
Number of Days of Vomiting or Nausea	s = 0.65 p = < 0.0001	s = 0.19 *p* = 0.09	s = 0.26 *p* = 0.02
Number of Vomiting Episodes	s = 0.38 *p* = 0.0004	s = 0.09 *p* = 0.43	s = 0.21 *p* = 0.06
Mean Severity of Nausea	s = 0.67 p = < 0.0001	s = 0.06 *p* = 0.15	s = 0.31 *p* = 0.005

s = Spearman correlation

Hypotheses about discriminant validity were confirmed. Spearman correlations between NVSA^©^ scores and KDQOL-36™ shortness of breath and faintness bothersomeness ranged from 0.06 to 0.31 (mean and median of 0.19 and 0.20, respectively); five of the six discriminant correlations met criteria for a small correlation (Table 4). Six of the 15 (40%) of the Spearman discriminant correlations between NVSA© scores and EQ-5D-5 L dimensions were statistically significant at conventional probability levels (*p* ≤ 0.05). These discriminant correlations ranged from − 0.06 to 0.42 (mean and median of 0.19 and 0.20, respectively); 11 of the 15 discriminant correlations (73%) met criteria for a small correlation (Table 5). Spearman correlations of the NVSA^©^ scores with the EQ-5D-5 L VAS were all statistically significant and ranged from − 0.24 to − 0.33 (mean and median of − 0.28); two of the three discriminant correlations met criteria for a small correlation (Table 5).

**Table 5:** Assessment of discriminant validity of NVSA^©^ scores with the EQ-5D-5 L: Week 1

	EQ-5D-5 L Dimensions					
NVSA©	Mobility	Self-care	Usual activities	Pain/ discomfort	Anxiety/ depression	VAS
Number of Days or Vomiting or Nausea	s = 0.09 *p* = 0.40	s = 0.19 *p* = 0.08	s = 0.21 *p* = 0.06	s = 0.36 *p* = 0.0009	s = 0.39 *p* = 0.0003	s = − 0.28 *p* = 0.01
Number of Vomiting Episodes	s = −0.06 *p* = 0.59	s = − 0.01 *p* = 0.93	s = 0.11 *p* = 0.34	s = 0.22 *p* = 0.05	s = 0.27 *p* = 0.01	s = − 0.24 *p* = 0.03
Mean Severity of Nausea	s = 0.08 *p* = 0.50	s = 0.15 *p* = 0.19	s = 0.20 *p* = 0.07	s = 0.42 *p* < 0.0001	s = 0.36 p = 0.0009	s = − 0.33 *p* = 0.003

s = Spearman correlation

#### Known-groups validity

Each of the nine tests of known-groups validity were statistically significant: there was a statistically higher number of days of vomiting or nausea per week, number of episodes of vomiting per week, and mean severity of nausea for subjects with vomiting/nausea as defined by the external criteria (Table 6). Number of days of vomiting or nausea per week and mean severity of nausea had similar discriminating ability to one another for three externally-defined known-groups while number of episodes of vomiting per week bore a somewhat weaker relationship to the known groups.

**Table 6:** Assessment of known-groups validity for NVSA^©^ scores

	n	Presence Mean (SE)	Absence Mean (SE)	Difference in Means (Presence-Absence)	Wilcoxon Two-Sample Test	*P* Value
sPGA^a^						
Number of Days of Vomiting or Nausea	78	4.16 (0.40)	0.61 (0.21)	3.55	6.57	< 0.0001
Number of Episodes of Vomiting	78	1.88 (0.41)	0.41 (0.15)	1.47	3.58	0.0002
Mean Severity of Nausea	78	1.81 (0.27)	0.13 (0.05)	1.68	6.69	< 0.0001
KDQOL-36™^b^						
Number of Days of Vomiting or Nausea	83	3.48 (0.39)	0.63 (0.22)	2.85	5.92	< 0.0001
Number of Episodes of Vomiting	83	1.39 (0.30)	0.53 (0.22)	0.86	2.97	0.0015
Mean Severity of Nausea	83	1.39 (0.23)	0.20 (0.08)	1.19	5.93	< 0.0001
FLIE^c^						
Number of Days of Vomiting or Nausea	83	3.61 (0.44)	0.78 (0.20)	2.83	5.39	< 0.0001
Number of Episodes of Vomiting	83	1.49 (0.33)	0.54 (0.20)	0.95	3.11	0.0009
Mean Severity of Nausea	83	1.50 (0.26)	0.22 (0.08)	1.28	5.69	< 0.0001

*N* sample size, *SE* standard error

^a^Presence: sPGA nausea ˃ 1; Absence: sPGA nausea = 1

^b^Presence: KDQOL-36™ nausea bothersomeness > 1; Absence: KDQOL-36™ nausea bothersomeness =1 nausea. Because NVSA© diary completion started at enrollment (study week 0), week 1 NVSA© data (enrollment to week 1) were used for this analysis while KDQOL-36™ reflects enrollment (week 0)

^c^Presence: FLIE Total < 108; Absence: FLIE Total ≥ 108. Because NVSA© diary completion started at enrollment (study week 0), week 1 NVSA^©^ data (enrollment to week 1) were used for this analysis while FLIE reflects enrollment (week 0)

#### Ability to detect change

The Spearman correlation between the eight weekly sPGA change scores and NVSA^©^ change scores ranged from 0.14 to 0.43 for number of days of vomiting or nausea per week (mean and median of 0.28 and 0.29, respectively), 0.09 to 0.58 for number of episodes of vomiting per week (mean and median of 0.28 and 0.29, respectively), and 0.14 to 0.49 for mean severity of nausea (mean and median of 0.34 and 0.38, respectively). Even though a small handful of the 21 calculated correlations were small―and perhaps meaningless (i.e., the single observed correlation of 0.09)―a broader gestalt view of the 21 correlations suggested that, by standard criteria [42, 43], the sPGA represents a marginally-acceptable criteria for assessing ability to detect change in the NVSA^©^ scores. Because of the small sample size and the sparse distributions of the sPGA’s, some statistical comparisons were not effected (blank cells in Appendix).

Table 7 presents a summary of results for ability to detect change (detailed results by week are presented in Appendix). Consistent with expectations, mean differences for the no-change group for all three NVSA^©^ scores were small (mean and median, respectively: 0.02 and − 0.00 for number of days of vomiting or nausea; 0.06 and 0.07 for number of vomiting episodes, and 0.00 and 0.04 for mean severity of nausea). Effect sizes for the no-change group for all three NVSA^©^ scores were small (all less than 0.13).

**Table 7:** Summary of results of ability to detect change of NVSA^©^ scores with the sPGA and effect size

	No change			Any worsening			Any improvement		
	Number of days of vomiting or Nausea	Number of vomiting episodes	Mean severity of Nausea	Number of days of vomiting or Nausea	Number of vomiting episodes	Mean severity of Nausea	Number of days of vomiting or Nausea	Number of vomiting Episodes	Mean severity of Nausea
Difference in Means									
Range	−0.13 – 0.19	− 0.15 – 0.51	− 0.12 – 0.21	− 0.05 – 1.83	− 0.99 – 2.40	− 0.22 – 0.76	− 1.80 – 0.17	−3.77 – − 1.07	−1.02 – − 0.21
Mean	0.02	0.06	0.00	0.65	1.21	0.32	− 0.88	−2.14	0.49
Median	0.00	0.07	0.04	0.55	0.21	0.45	−1.05	−1.37	−0.29
Within-Patient Effect Size									
Range	0.00–0.08	0.00–0.29	0.01–0.17	0.02–0.78	0.26–1.37	0.06–0.73	0.07–0.73	0.60–0.97	0.22–0.82
Mean	0.04	0.12	0.06	0.28	0.88	0.36	0.38	0.83	0.40
Median	0.04	0.07	0.04	0.23	0.60	0.38	0.42	0.88	0.29

The within-patient effect size was defined as the within-patient change score (post-pre score) divided by pre (group) standard deviation

**Table 8:** Distribution-based definitions of meaningful change for NVSA^©^ scores

	Number of days of vomiting or Nausea	Number of episodes of vomiting	Mean severity of Nausea
0.50 Standard Deviation			
Week 1	1.23	0.87	0.62
Week 2	1.17	1.94	0.70
Week 3	1.18	0.94	0.47
Week 4	1.17	1.05	0.52
Week 5	1.24	0.75	0.56
Week 6	1.24	1.06	0.62
Week 7	1.26	0.89	0.50
Week 8	1.07	1.17	0.41
Mean	1.19	1.08	0.55
Median	1.20	0.99	0.54
One SEM	0.63	1.35	0.27

*SEM* standard error of measurement

Across the three NVSA^©^ scores, 75% of the mean differences for the worsening group were positive in direction as expected. For number of days of vomiting or nausea, the mean and median mean difference was 0.65 and 0.55, respectively, and mean and median effect sizes were 0.28 and 0.23, respectively. The mean and median effect size for number of vomiting episodes was 0.88 and 0.60, respectively, and the same for mean severity of nausea was 0.36 and 0.38, respectively.

Across the three NVSA^©^ scores, 94% of the mean differences for the improvement group were negative as expected. For number of days of vomiting or nausea, the mean and median mean difference was − 0.88 and − 1.05, respectively, and mean and median effect sizes were 0.38 and 0.42, respectively. The mean and median effect sizes for number of vomiting episodes was 0.83 and 0.88, respectively, and the same for mean severity of nausea was 0.49 and 0.29, respectively.

#### Minimal meaningful change thresholds

##### Distribution-based results

The mean and median one-half SD for number of days of vomiting or nausea was 1.19 and 1.20, respectively (Table 8). The mean and median one-half SD for number of episodes of vomiting was 1.08 and 0.99, respectively, and the same for mean severity of nausea was 0.55 and 0.54, respectively***.*** The one SEM for number of days of vomiting and nausea was 0.63, 1.35 for number of episodes of vomiting, and 0.27 for mean severity of nausea***.***

##### Anchor-based results using the sPGA

Because of the small sample size and the sparse distributions of the sPGA change scores, 52% of the analyses could not be executed. Because a small sample of subjects contributed data to these analyses and because two observed results were anomalous (positive change for minimally improve for number of days of vomiting and nausea and negative change for minimally worsen for mean severity of nausea), a single, preliminary threshold for minimal-meaningful change is proposed rather than a range of thresholds. Thresholds for both minimal worsening and improvement are presented for comprehensiveness sake (Table 9).

**Table 9:** Anchor-based assessment of meaningful change for NVSA^©^ scores with sPGA

	Number of Days of Vomiting or Nausea	Number of Episodes of Vomiting	Mean Severity of Nausea
sPGA Score Used	n, mean Δ between weeks, SE Δ	n, mean Δ between weeks, SE Δ	n, mean Δ between weeks, SE Δ
	Minimally Worsen	Minimally Worsen	Minimally Worsen
Week 2-Week 1	(*n* = 7) 0.68 (0.50)		(*n* = 7) 0.31 (0.16)
Week 3-Week 2	(*n* = 7) 0.58 (0.86)		(*n* = 7) −0.39 (0.78)
Week 4-Week 3			
Week 5-Week 4			
Week 6-Week 5	(*n* = 8) 0.59 (0.57)	(*n* = 6) 1.42 (0.92)	(*n* = 8) 0.23 (0.10)
Week 7-Week 6	(*n* = 6) 0.17 (0.70)		(*n* = 6) 0.48 (0.34)
Week 8-Week 7			
Mean	0.50	1.42	0.16
Median	0.58	1.42	0.27
	Minimally Improve	Minimally Improve	Minimally Improve
Week 2-Week 1	(*n* = 7) −1.80 (0.92)		(*n* = 7) −1.02 (0.47)
Week 3-Week 2	(*n* = 7) 0.11 (0.84)	(*n* = 6) −1.03 (0.35)	(*n* = 7) −0.46 (0.24)
Week 4-Week 3	(*n* = 6) − 1.07 (0.52)		(*n* = 6) − 0.15 (0.16)
Week 5-Week 4			
Week 6-Week 5			
Week 7-Week 6	(*n* = 7) − 0.68 (0.68)	(*n* = 7) −1.64 (1.02)	(*n* = 7) − 0.22 (0.21)
Week 8-Week 7			
Mean	−0.86	−1.33	−0.46
Median	−0.87	−1.33	−0.34

Empty rows = sample size < *n* = 6 for minimally improve or minimally worsen and analyses not conducted

*n* = sample size; Δ = change score; SE = standard error

For number of days of vomiting or nausea, sample size permitted four tests. The minimally-worsened group had positive change scores (mean and median of 0.50 and 0.58, respectively). The minimally-improved group had both negative and positive change scores (mean and median of − 0.86 and − 0.87, respectively). For number of episodes of vomiting, sample size permitted three tests. The one minimally-worsened group had a change score of 1.42. The minimally-improved group had negative change scores (mean and median both − 1.33).

For mean severity of nausea, the minimally-worsened group had positive and negative change scores (mean and median of 0.16 and 0.27, respectively). The minimally-improved group had negative change scores (mean and median of − 0.46 and − 0.34, respectively).

##### Triangulation between distribution- and anchor-based results

According to the FDA [11], “distribution-based methods for determining clinical significance of particular score changes should be considered as supportive and are not appropriate as the sole basis for determining a responder definition.” Accordingly, triangulation is used to examine multiple values from different approaches to converge on a small range of values [42, 56, 57]. Thresholds for meaningful change should exceed the bounds of measurement error, i.e., distribution-based approaches should define the lower limits of meaningful-change thresholds [38, 58]. In the case of the NVSA,^©^ however, the anchor-based estimates were smaller than the distribution-based estimates for number of days with nausea and vomiting and mean severity of nausea. Therefore, the following preliminary thresholds for meaningful worsening are suggested based upon interpolation between the 0.50 SD and one SEM: 0.90 for number of days of nausea and vomiting, 1.20 for number of vomiting episodes; and 0.40 for mean severity of nausea. These estimates should be considered strictly provisional until additional interventional, longitudinal research has been completed.

## Discussion

We described herein the development and psychometric validation of the NVSA^©^―a new PRO instrument that measures nausea and vomiting among CKD patients on maintenance hemodialysis with SHPT. The development of the NVSA^©^ began with a review of information from clinical studies and expert clinical guidelines. These data supported nausea and vomiting as important adverse reactions associated with oral calcimimetic cinacalcet. As the FDA PRO Guidance [11] states: “[i]n some cases, the question of what to measure may be obvious given the condition being treated,” and this was squarely the case with CKD patients on maintenance hemodialysis with SHPT treated with oral calcimimetic cinacalcet. Traditional cognitive-debriefing interviews revealed that patients understood the NVSA^©^ instructions, items, and response scales (although additional training was recommended and implemented). A full 73% of patients described the eDiary as “very easy” or “quite easy” to use, and 100% of patients described the eDiary as “acceptable to use.” Confirmatory concept-elicitation interviews cross-validated the data from clinical studies and expert clinical guidelines; these interviews confirmed that the NVSA^©^ captures relevant aspects of nausea (severity) and vomiting (frequency) from the patient point of view.

The psychometric analyses support most measurement characteristics of the NVSA^©^. The NVSA^©^ scores correlated significantly with the FLIE scales and KDQOL-36™ nausea item and exhibited markedly smaller correlations with KDQOL-36™ shortness of breath and faintness and the EQ-5D-5 L dimensions and VAS. Tests of known-groups validity were highly supportive of the NVSA^©^ with the group defined by the presence of nausea/vomiting having statistically higher mean NVSA^©^ scores than the absence group. Test-retest reliability was excellent for number of days of nausea and vomiting and mean severity of nausea. Test-reteat reliability was somewhat smaller for number of vomiting episodes likely because few subjects reported vomiting episodes (lack of between-subjects variability).

The pattern of results for ability to detect change was as expected. The no-change group showed little change and had effect sizes close to zero. Mean differences for the worsening group were mostly positive as expected and the same for the improvement group were mostly negative as expected. With a few exceptions, the mean and median effect sizes for the worsening and improvement groups met standard criteria for medium or large effect sizes. That the worsening and improvement groups exhibited larger mean difference and effect sizes than the no-change group provides evidence of face validity of the ability-to-detect-change analyses. These analyses were limited by the small sample size overall and the fact that a large proportion of subjects in the non-interventional, observational study changed little over time. Future research is necessary to replicate these analysis in larger samples undergoing treatment.

Preliminary minimal meaningful change thresholds were an increase of 0.90 days with vomiting or nausea per week, 1.20 vomiting episodes per week, and 0.40 for mean severity of nausea. These estimates were based on group-level data. Given that vomiting and nausea are burdensome experiences, small changes in NVSA^©^ scores could be considered clinically meaningful for CKD patients with SHPT. Definitions of minimal meaningful change are not a static or fixed attribute of a PRO instrument and evolve as more evidence is gathered through empirical studies. [56] Additional interventional research is necessary to recommend more robust thresholds for meaningful change.

Our study has several limitations. The initial items and response categories were drafted by a cross-functional expert team that included two health outcomes/PRO researchers, one medical development lead, and one safety/regulatory lead. External clinical experts were not involved in the initial PRO development. The ten patients recruited and successfully interviewed for the confirmatory concept-elicitation interviews had completed the eight-week data collection period for the non-interventional, observational study; as such they may have been sensitized to topics covered in the non-interventional, observational study data-collection protocol. However, the intent of the concept-elicitation interviews was to be confirmatory in nature―to identify any potential outcomes *aside from nausea and vomiting*―not identified by the review of information from clinical studies [8–10, 17–19] and expert clinical guidelines [20]. Although the most frequently-reported adverse reactions for cinacalcet are nausea and vomiting, less than 15% of the sample were subjects who would have been expected to experience new episodes of nausea/vomiting due to initiation of cinacalcet. This reduced the variability in the NVSA^©^ scores: almost half of the sample reported no nausea/vomiting during the first study week (floor effects) thus preventing analysis of improvement in scores over time. It is, however, ideal for capturing worsening. Due to the non-interventional nature of the study, neither substantial improvement nor worsening was expected to occur. The small sample limited tests of ability to detect any improvement or worsening (since most of the sample reported no change on the sPGA). With eight sPGA’s across 8 weeks, there was an endless number of possible combinations and permutations to assess ability to detect change and meaningful change―none of which have any innate conceptual, methodological, and psychometric superiority over one another. Because we were contending with small sample sizes, we utilized an approach to garner “strength across numbers.” By utilizing all 8 weeks, we derived multiple estimates that could be summarized by measures of central tendency instead of a more limited approach of arbitrarily selecting a handful of weeks for analysis. By utilizing all 8 weeks, it was also possible to visually assess the consistency of within-patient change across the 8 weeks and across definitions of worsening, no change, and improvement which added confidence to the interpretation of the data despite the small sample size. Derivation of the anchor-based thresholds of meaningful change might have been more conclusive if sample size was larger for the minimally-improved and minimally-worsened groups. Additional assessment of the performace of the NVSA^©^, particularly thresholds for meaningful change, in the context of any clinical trial that it may be used in are warrented.

## Conclusions

Tolerability issues can impact adherence with medications and, ultimately, treatment efficacy. Nausea and vomiting are the most commonly-reported adverse reactions experienced by patients taking the oral calcimimetic cinacalcet for SHPT associated with CKD requiring dialysis. Developing a reliable and valid measure of nausea/vomiting in this population will facilitate the measurement of these reactions in clinical trials. This study supported many of the psychometric characteristics of a short daily diary designed to measure nausea and vomiting. The NVSA^©^ may be used to support study endpoints in clinical trials comparing the nausea and vomiting profile of novel SHPT therapies.

## Appendix

**Table 10:** Assessment of ability to detect change of NVSA^©^ scores with the sPGA and effect size

		Number of days of vomiting or Nausea			Number of vomiting episodes			Mean severity of Nausea		
sPGA Week	sPGA	N	Difference in means	Effect Size	N	Difference in means	Effect size	N	Difference in means	Effect size
(Week 2 – Week 1)	No Change	41	0.19 (0.98)	0.08	42	0.51 (2.49)	0.29	41	0.21 (0.93)	0.17
(Week 3 – Week 2)	No Change	44	−0.10 (0.97)	0.04	47	−0.01 (1.43)	0.00	44	−0.05 (0.74)	0.04
(Week 4 – Week 3)	No Change	52	0.15 (1.10)	0.06				52	−0.01 (0.50)	0.01
(Week 5 – Week 4)	No Change	47	−0.00 (0.95)	0.00	49	−0.13 (1.18)	0.06	47	−0.04 (0.35)	0.04
(Week 6 – Week 5)	No Change	45	0.07 (1.13)	0.03	53	0.26 (1.24)	0.17	45	0.03 (0.40)	0.03
(Week 7 – Week 6)	No Change	44	−0.01 (0.77)	0.00	47	−0.15 (0.75)	0.07	44	−0.04 (0.30)	0.03
(Week 8 – Week 7)	No Change	47	−0.13 (1.09)	0.05	49	−0.14 (0.77)	0.08	47	−0.12 (0.38)	0.12
(Week 2 – Week 1)	Any Improvement	7	−1.80 (2.43)	0.73				7	−1.02 (1.24)	0.82
(Week 3 – Week 2)	Any Improvement	9	−0.47 (2.37)	0.20	8	−3.77 (8.21)	0.97	9	−0.74 (1.11)	0.53
(Week 4 – Week 3)	Any Improvement	7	−1.06 (1.16)	0.45				7	−0.21 (0.39)	0.22
(Week 5 – Week 4)	Any Improvement	7	−1.26 (2.56)	0.54	6	−1.67 (2.73)	0.79	7	−0.26 (1.65)	0.25
(Week 6 – Week 5)	Any Improvement	7	0.17 (2.52)	0.07				7	−0.26 (0.69)	0.23
(Week 7 – Week 6)	Any Improvement	10	−0.67 (1.56)	0.27	8	−2.06 (2.76)	0.97	10	−0.62 (1.46)	0.49
(Week 8 – Week 7)	Any Improvement	7	−1.05 (1.30)	0.42	7	−1.07 (6.47)	0.60	7	−0.29 (0.48)	0.29
(Week 2 – Week 1)	Any Worsening	10	0.55 (1.99)	0.22	11	2.40 (7.44)	1.37	10	0.54 (1.05)	0.44
(Week 3 – Week 2)	Any Worsening	9	0.56 (1.99)	0.24	7	−0.99 (4.63)	0.26	9	− 0.22 (1.85)	0.16
(Week 4 – Week 3)	Any Worsening									
(Week 5 – Week 4)	Any Worsening	6	1.83 (1.72)	0.78				6	0.76 (0.78)	0.73
(Week 6 – Week 5)	Any Worsening	11	0.70 (1.56	0.28	6	1.42 (2.25)	0.95	11	0.40 (0.56)	0.36
(Week 7 – Week 6)	Any Worsening	8	0.33 (1.55)	0.13	7	2.00 (2.58)	0.94	8	0.51 (0.72)	0.41
(Week 8 – Week 7)	Any Worsening	6	−0.05 (0.88)	0.02				6	−0.06 (1.13)	0.06

Empty rows = sample size < n = 6 and analyses not conducted. The within-patient effect size was defined as the within-patient change score (post-pre score) divided by pre (group) standard deviation

## Abbreviations

CKD: Chronic kidney disease
eDiary: electronic diary
EQ-5D-5 L: European Quality of Life 5 Dimension 5 Level
ERT: eResearch Technologies
FDA: Food and Drug Administration
FLIE: Functional Living Index-Emesis
HRQoL: Health-related quality of life
ICC: Intraclass correlation coefficient
IRB: Institutional review board
KDQOL-36™: Kidney Disease Quality of Life Instrument
N: Sample size
NVSA: Nausea/Vomiting Symptom Assessment
PGRC: Patient Global Rating of Change
PRO: Patient-reported outcome
SD: Standard deviation
SE: Standard error
SEM: Standard error of measurement
SHPT: Secondary hyperparathyroidism
sPGA: static Patient Global Assessment
VAS: Visual analogue scale
